# ER stress regulates alkaline phosphatase gene expression in vascular smooth muscle cells via an ATF4-dependent mechanism

**DOI:** 10.1186/s13104-018-3582-4

**Published:** 2018-07-16

**Authors:** Malgorzata Furmanik, Catherine M. Shanahan

**Affiliations:** 1 Cardiovascular Division, James Black Centre, King’s College London, 125 Coldharbour Lane, London, SE5 9NU UK; 2 Present Address: Department of Biochemistry, CARIM-Cardiovascular Research Institute Maastricht, Maastricht University, Maastricht, The Netherlands

**Keywords:** Alkaline phosphatase, ATF4, Promoter, Vascular smooth muscle cell, Endoplasmic reticulum stress

## Abstract

**Objective:**

Vascular calcification is the deposition of hydroxyapatite crystals in the blood vessel wall. Osteogenic differentiation of vascular smooth muscle cells (VSMCs) plays a key role in this process. Increased expression of alkaline phosphatase (ALP) occurs in some in vitro models of VSMC calcification and is thought to be crucial for mineralization, however, little is known about the transcriptional regulation of ALP in VSMCs. Recently, ALP upregulation was shown to coincide with endoplasmic reticulum (ER) stress-mediated vascular calcification, specifically with expression of the transcription factor ATF4. As no direct links between ALP expression and ER stress have previously been demonstrated in VSMCs, the aim of this study was to investigate whether ATF4 interacts directly with the ALP promoter.

**Results:**

The present study shows that ALP mRNA and activity were significantly increased by ER stress treatment of human primary VSMCs in vitro and that this was ATF4-dependent. Bioinformatics analysis predicted two ATF4 binding sites in ER-stress responsive regions of the ALP promoter (− 3631 to − 2048 bp from the first intron). However, we found that ATF4 does not bind within this fragment of the ALP promoter region.

**Electronic supplementary material:**

The online version of this article (10.1186/s13104-018-3582-4) contains supplementary material, which is available to authorized users.

## Introduction

Vascular calcification is the deposition of hydroxyapatite crystals in the blood vessel wall [1, 2]. The presence of vascular calcification results in vascular stiffness and increased risk of cardiovascular and all-cause mortality. Vascular smooth muscle cells (VSMCs) play a key role in regulating vascular calcification via processes that have also been implicated in bone formation [3] including apoptosis [4], release of exosomes [5], loss of calcification inhibitors [6] and osteogenic differentiation [7].

Alkaline phosphatase (ALP, TNAP, ALPL), a key inducer of mineralization in bone, catalyses dephosphorylation of pyrophosphate resulting in inactivation of this calcification inhibitor and providing phosphate ions for hydroxyapatite crystal formation. ALP is ubiquitously expressed in tissues and increased levels in serum and the vasculature have been linked to vascular calcification [8, 9]. Increased expression of ALP also occurs in some in vitro models of VSMC calcification where it is thought to be crucial for mineralisation [10–12]. Previous studies identified transcription factor binding motifs on the human ALP promoter (TATA box, Sp1 binding site [13], vitamin D response element-like motifs, TATA boxes, E-box-like sequences, Sp1 binding site [14]) and mouse promoter (enhancer sequence and E-box [15]). Additionally, several transcription factors (DIF-1 [16], forkhead transcription factor FKHR [17], Dlx5 [18] and p107 retinoblastoma family transcription factor [19]) have been shown to bind the ALP promoter and regulate ALP expression in osteoblasts. Despite this, little is known about the regulation of this enzyme in VSMCs.

The endoplasmic reticulum (ER) is an organelle where secreted and transmembrane proteins are folded and mature. ER stress occurs when the influx of unfolded proteins to the ER is larger than its capacity to fold them, resulting in activation of a signalling pathway called the unfolded protein response (UPR) [20]. The UPR is mediated by ER stress transducers: IRE1, ATF6 and PERK. Each of these transducers activates a distinct signalling pathway, which together comprise the UPR. Transcription factor AFT4 is preferentially translated when PERK is activated. Importantly, ER stress and the UPR have been shown to be crucial for bone development [21–25]. More recently, ALP upregulation has been shown to coincide with ER stress-mediated vascular calcification and ALP expression and activity levels have been shown to be ATF4-dependent in calcifying VSMCs  [11, 12, 26, 27]. Therefore, in the present study we set out to investigate whether there is a direct interaction of ATF4 with the ALP promoter in response to ER stress.

## Main text

### Materials and methods

Expanded “Materials and Methods” are available in Additional file 1.

### Cell culture and treatments

Human primary VSMCs were of aortic origin, collected from an adult donor with local ethics committee approval (Cambridge Local Research Ethics Committee LREC 97/084), characterised and archived in the laboratory. All human materials were handled in compliance with the Human Tissue Act (2004, UK). Cells were treated with 0.2 μg/ml thapsigargin (TG, Sigma, T9033) or 0.4 μg/ml tunicamycin (TM, Sigma, T7765). SiRNA gene knockdown was carried out using HiPerfect (Qiagen) and 3 pmol siRNA oligonucleotide smartpool (GE Dharmacon).

### Gene expression analysis

Gene expression was analysed with quantitative real time PCR (ALP F: ACGAGCTGAACAGGAACAACGT R: CACCAGCAAGAAGAAGCCTTTG, ATF4 F: CAACAACAGCAAGGAGGATGCCTT R: TGTCATCCAACGTGGTCAGAAGGT, GAPDH F: CGACCACTTTGTCAAGCTC R: CAAGGGGTCTACATGGCAAC) and western blotting (anti-ATF4, Santa Cruz, sc-200 and anti-Rabbit IRDye680 RD, Li-Cor, 926-68071).

### Alkaline phosphatase activity assay

ALP activity was measured in cell lysates colorimentrically at 405 nm using pNPP substrate (Sigma), normalised to protein concentration and expressed as μM of pNP generated per minute.

### Promoter analysis

The sequence corresponding to the − 122 to − 4556 (counting from the first nucleotide of the initiation codon) of the ALP promoter was analysed with Matinspector (Genomatix) for the presence of transcription factor binding sites. ALP promoter constructs [14] and pRL-TK renilla luciferase vector (Promega) were transfected into VSMCs using Lipofectamine LTX. Luciferase assays were carried out using the Dual-Luciferase Reporter Assay kit (Promega). DNA binding assays using nuclear extracts were performed as described previously [28], using biotinylated forward and normal reverse primers (F: GGAGTGTAGTGGCGTGATCT, R: GCAATAGAGTGGGACCCTGT).

### Mass spectrometry

Samples were analysed using liquid chromatography–tandem mass spectrometry (LC–MS/MS). Raw mass spectrometry data were analysed in Proteome Discoverer (ThermoScientific; v1.3.0.339) utilising the Mascot database. Samples were searched against Uniprot database to identify proteins.

### Data analysis

All results represent 3 independent experiments, unless stated otherwise. Graphs show mean with SEM. Where appropriate t-tests or one way ANOVA with Tukey’s or Dunnett’s post hoc tests were performed. Statistical significance is indicated with asterisks: * denotes p between 0.05 and 0.01, ** denotes p between 0.01 and 0.001, *** denotes p < 0.001.

### Results

#### ALP expression and activity are ATF4-dependent

**Fig. 1:**
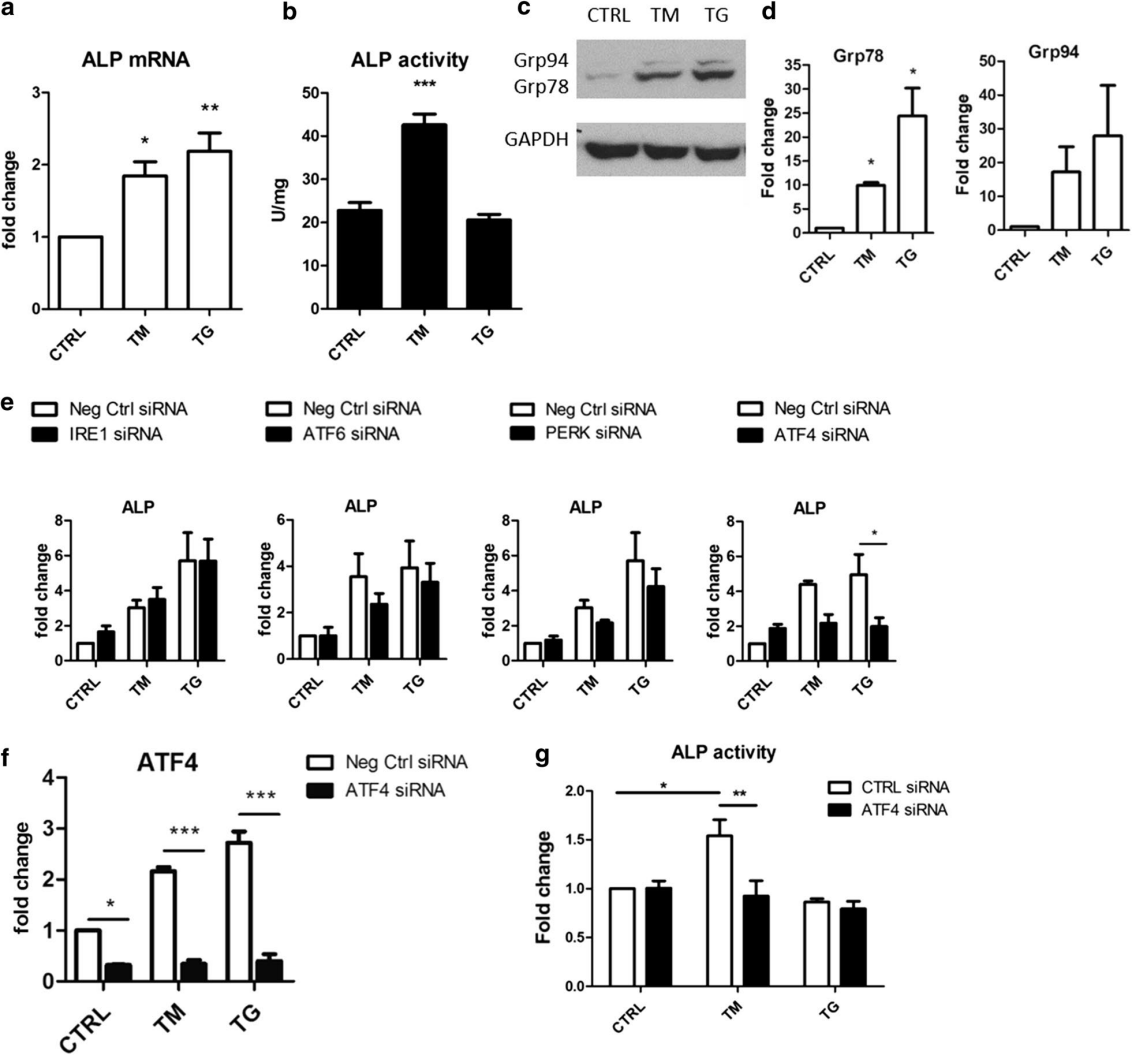
ALP expression and activity are ATF4-dependent in VSMCs. VSMCs were treated with 0.4 μg/ml TM or 0.2 μg/ml TG for 24 h. **a** ALP mRNA levels measured by qPCR. **b** ALP activity. **c**, **d** Western blotting for Grp78 and Grp94 chaperones indicated that ER stress was activated. **e** QPCR analysis of ALP mRNA expression in VSMCs treated with IRE1, ATF6, PERK or ATF4 siRNA or non-targeting siRNA (Neg Ctrl) and then TM or TG for 24 h. **f** QPCR analysis of ATF4 mRNA expression in VSMCs treated with ATF4 siRNA or non-targeting siRNA. **g** ALP activity in VSMCs treated with ATF4 siRNA or non-targeting siRNA. * p < 0.05, ** p < 0.01, *** p < 0.001

ALP mRNA levels were upregulated in VSMCs treated with ER stress inducers tunicamycin (TM) and thapsigargin (TG) (Fig. 1a). ER stress activation was confirmed by western blotting for ER chaperones Grp78 and Grp94 (Fig. 1c, d). ALP activity was significantly increased (twofold) by tunicamycin treatment, but did not change with thapsigargin treatment (Fig. 1b), suggesting only tunicamycin triggered signalling events that lead to the activation of the enzyme.

SiRNA knock-down of IRE1, PERK and ATF6 caused small, but insignificant decreases in ALP expression. In contrast, knock-down of ATF4 caused a twofold decrease in ALP mRNA expression (Fig. 1e, f) corresponding to a decrease in ALP activity (Fig. 1g). ATF4 knock-down also blocked the increase in ALP activity induced by tunicamycin but had no effect on activity at baseline or in thapsigargin-treated cells.

#### ER stress induces ALP promoter activity

**Fig. 2:**
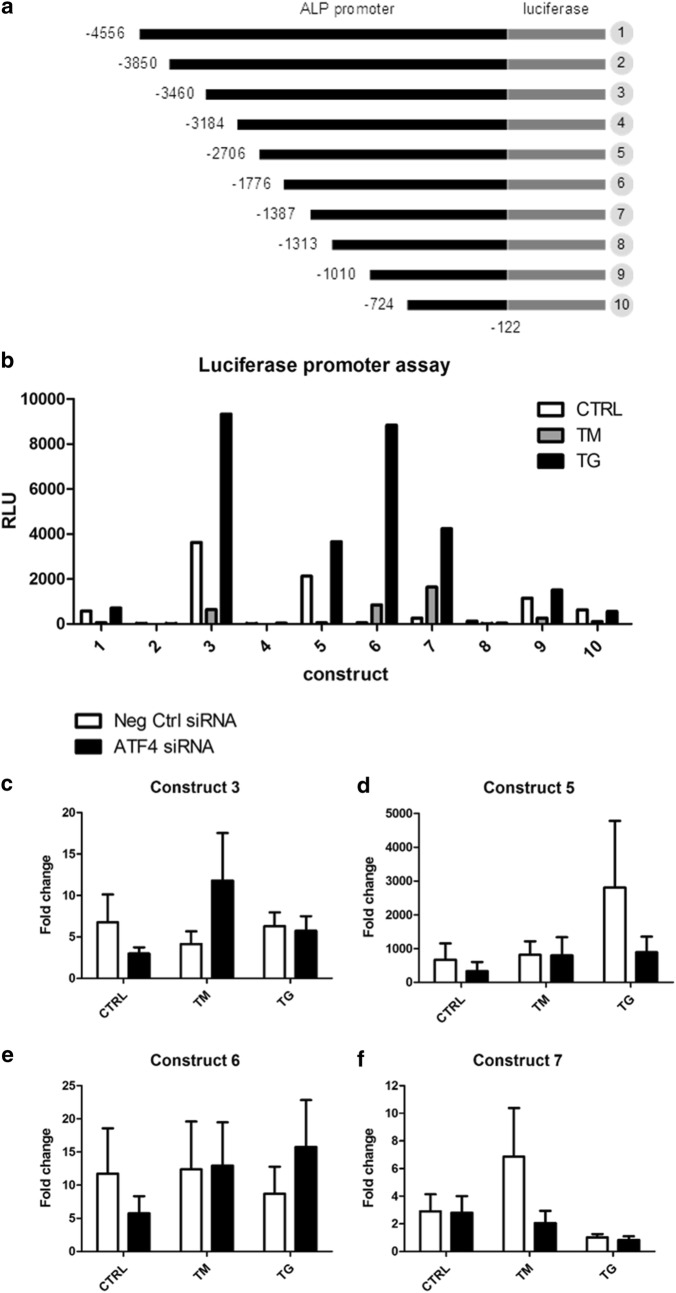
ALP promoter activity in VSMCs in response to ER stress and ATF4 knock-down. **a** Schematic showing ALP promoter constructs for luciferase assay. Construct 1 is the full length fragment, constructs 2–10 were derived by deleting increasing portions of the full length. Based on [14]. **b** Activity of luciferase expressed from ALP promoter constructs in VSMCs, n = 1. VSMCs were transfected with constructs for 24 h and then treated with 0.4 μg/ml TM or 0.2 μg/ml TG for 24 h. **c**–**f** Activity of luciferase expressed from ALP promoter constructs 3, 5, 6 and 7 in VSMCs treated with ATF4 siRNA or non-targeting siRNA, n = 3

To determine whether ALP promoter activity was regulated by ER stress, luciferase assays were performed with 10 constructs containing fragments of the 5′-upstream region of the human ALP gene spanning from − 122 to − 4556 (Fig. 2a, the full annotated sequence can be found in Additional file 1: Figure S1). Results showed that constructs 1, 3, 5, 6, 7, 9 and 10 contained regions of the ALP promoter that were transcriptionally active in VSMCs and luciferase was activated further in constructs 3, 5, 6 and 7 by thapsigargin treatment, and construct 6 and 7 also by tunicamycin (Fig. 2b).

To examine whether ATF4 plays a direct role in activation of these responsive fragments luciferase assays were performed with simultaneous ATF4 siRNA knock-down. Despite achieving consistent knock-down, the results of the luciferase assays were inconclusive with no statistically significant differences (Fig. 2c–f).

#### ALP promoter contains ER stress-related transcription factor binding sites

**Fig. 3:**
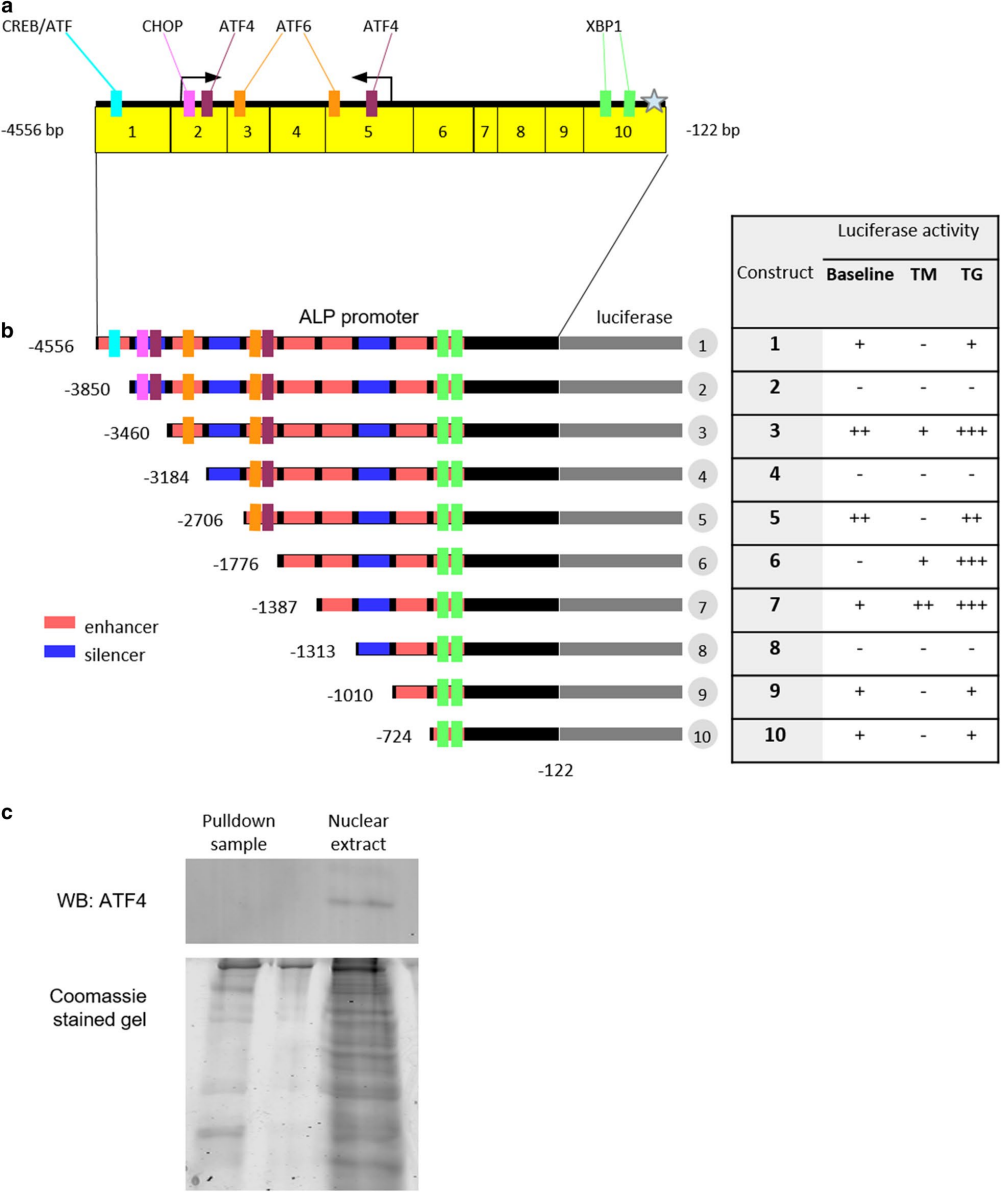
ATF4 does not bind the ALP promoter in VSMCs. **a** ER stress-related transcription factor binding sites mapped to the ALP promoter. **b** Schematic of ALP promoter constructs with potential regions activating (red) and inhibiting (blue) transcription in VSCMs, based on luciferase assay results. ‘+’ denotes degrees of activation, ‘−’ denotes lack of promoter activity. **c** The fragment of the ALP promoter containing potential ATF4 binding sites was amplified using biotinylated primers and conjugated onto magnetic streptavidin beads. The beads were then incubated with VSMC nuclear extracts, washed and proteins were eluted off the DNA and analysed by Western blotting. Coomassie gel demonstrates that even though ATF4 was not detected in the pulldown sample, other proteins were present. N = 3, figure shows representative images

Transcription is regulated by both positive and negative factors and based on the luciferase assays it was possible to infer which sequences activate (enhancers) and which suppress (silencers) transcription of the ALP promoter (Fig. 3b). Potential silencers were present in fragments 2, 4 and 8, surrounded by sequences that activate transcription while the promoter fragment shared by constructs 3–7 contained a sequence that was highly active in VSMCs and inducible by ER stress.

To examine known ER stress responsive consensus binding sites within the ALP promoter bioinformatics analysis with Genomatix Matinspector was performed. Several binding sites for ATF6, ATF4, CHOP and XBP1 were predicted in the full length sequence Fig. 3a and these sites were mapped onto the luciferase constructs (Fig. 3b). ATF6, XBP1, and CREB/ATF binding sequence were scattered in activator regions of various constructs. Two ATF4 binding sites were predicted, one localised in a silencer sequence and one in an enhancer, in a region encompassed by constructs 1–5. However, there was no clear enrichment of these sequences in fragments 3 and 7 that were ER stress responsive. This suggests that indirect interactions of ER stress-related transcription factors with other, directly binding, factors are responsible for increased activity in ER stress conditions.

#### ATF4 does not bind the ALP promoter

The promoter fragment containing both potential ATF4 binding sites (Fig. 3a, arrowed) was amplified using biotinylated primers, conjugated onto magnetic streptavidin beads and incubated with VSMC nuclear extracts. Western blotting of eluted proteins did not detect ATF4 (Fig. 3c) despite its presence in the start lane, suggesting ATF4 did not bind.

For more sensitive detection we subjected eluted proteins to liquid chromatography–mass spectrometry (LC–MS/MS) (Additional file 1: Figure S2). A thapsigargin-treated nuclear extract was used for the proteomic experiment to increase the chance of finding protein-ALP promoter interactions relevant to ER stress. The mass spectrometry analysis identified 447 different proteins with 95% or more probability, for which more than 1 peptide was present, in both lanes. Results indicate that no ATF4 was detected (Additional file 2) nor were any of the other predicted ER stress-related transcription factors.

### Discussion

This study shows that ALP mRNA and activity were significantly increased by ER stress in human primary VSMCs in vitro. siRNA knock-down showed that ATF4 is required for ER stress-induced ALP expression and luciferase reporter assays identified regions of the ALP promoter responsive to ER stress. Bioinformatics predicted two ATF4 binding sites within these fragments however DNA binding assays and MS failed to show ATF4 binding, suggesting ATF4 regulation is not via direct promoter interactions.

There are several possible explanations for this: (1) bioinformatics predictions may not be accurate, (2) ATF4 may not be a direct regulator of ALP expression, but could act via other, unknown downstream factors that were downregulated after ATF4 knock-down and (3) the ATF4-responsive element could be located outside the examined promoter fragment [29]. This is supported by the fact that tunicamycin caused an increase in ALP mRNA expression and enzyme activity levels, but did not consistently induce promoter activity.

Our results also suggest that tunicamycin and thapsigargin regulate ALP via different pathways and this is in line with studies showing that in VSMCs these two compounds can have differential effects on gene expression [30].

The proteomics results also suggest indirect regulation by ER stress. The responsive fragments contained a TCF/LEF binding site (data not shown, [16]) and β-catenin, which was identified by proteomics, activates transcription by forming a complex with TCF and LEF [31, 32]. β-Catenin belongs to the Wnt signalling pathway crucial for bone formation, and has been implicated in vascular calcification [33]. Importantly, ER stress has been shown to activate β-catenin in embryonic stem cells [34] suggesting that β-catenin could be a regulator of ALP expression in VSMCs in response to ER stress but this requires further testing.

#### Limitations

The main limitation of this study is the lack of consistent data for ALP promoter activity after ATF4 knock-down. However, using a direct approach of proteomics we did not find ATF4 binding the examined fragment of ALP promoter. It is also possible that ATF4 is a regulator of the ALP promoter, but our analysis did not encompass the right region of the promoter.

## Additional files

**Additional file 1: Figure S1.** The sequence of the ALP promoter with ER stress-related transcription factor binding sites. **Figure S2.** Coomassie gel used for proteomic analysis of proteins bound to the ALP promoter. Full description of materials and methods.

**Additional file 2.** Proteomics results. List of proteins, that bind the ALP promoter, identified by mass spectrometry. Nuclear extracts were bound and eluted off the biotinylated DNA on beads and analysed by LC–MS/MS. Raw mass spectrometry data were analysed in Proteome Discoverer (ThermoScientific; v1.3.0.339) utilising the Mascot database. Samples were searched against Uniprot database to identify proteins bound to the ALP promoter and compared with proteins that nonspecifically bound to unconjugated beads.

## Abbreviations

ALP: alkaline phosphatase
ATF4: activating transcription factor 4
ATF6: activating transcription factor 6
CHOP: C/EBP-homologous protein
ER: endoplasmic reticulum
IRE1: inositol-requiring enzyme 1
LC–MS/MS: liquid chromatography–tandem mass spectrometry
PERK: PRKR-Like Endoplasmic Reticulum Kinase
pNPP: p-nitrophenyl phosphate
TCF/LEF: T cell-specific transcription factor 1-alpha/lymphoid enhancer binding factor 1
TG: thapsigargin
TM: tunicamycin
UPR: unfolded protein response
VSMC: vascular smooth muscle cell
XBP1: X-box binding protein 1
