# Supplementary material for: ER stress regulates alkaline phosphatase gene expression in vascular smooth muscle cells via an ATF4-dependent mechanism
Source: BMC Res Notes. 2018 Jul 16;11:483. doi: 10.1186/s13104-018-3582-4 (PMC6048897; doi:10.1186/s13104-018-3582-4)
Supplement: Supplementary file 1 — Additional file 1: Figure S1. The sequence of the ALP promoter with ER stress-related transcription factor binding sites. Figure S2. Coomassie gel used for proteomic analysis of proteins bound to the ALP promoter. Full description of materials and methods. [file 13104_2018_3582_MOESM1_ESM.docx]

**Additional file 1**

1 ACTGGGATTA CAGGCGTGTG CCACTGTGCC CGGCCCCTGT TATCTTACTA ACTTACTTAT

61 TAATGTTTGT TGTTTATTAT CTACCTTCCC AACTAAAGTG TCAGTTCCGT GAGGATAAAC

121 ATTCTGTTTT GTTCACTGGT ATGTCCAGTT CCTAGAAGGG TGCCTGCTGC AGAGCAGGCA

181 TCCAATAAAC ATTTGTTGAA TGAATAAGGT TGCCAAGTCT GCCTGGGATA ACAGCCTGCT

241 CACTGGAAAG GTGACGATGA CAACAGTGAT GGTGCTTTGG TGTTGCAGGG AGGAGCAAGT

CREB/ATF

301 TAAATCTCAC CTATAAAGAT CTTTCCATCA GGCTGCAGAC ACAGAGGGAG TCCCCAGCAA

361 CAATAGCTCA TATATGCTTC AGTTTCCTCA TCCGTGAAGA GAGAATAAAA GTCCCTACTT

421 TTATTAGCGT CCAATTGCCC TGGCCACGGC AGCATTGTCG GTTTAATGAT GCTGCTTCGG

481 CTGTCGTAGT CTCTTCCACC TCATGCCTTT TGGTTCATTT TTTAACTGAG TTAAAGGTGG

541 GGCTGTAGGT GGCACTGGGA ATCAAATGGC TGAACTTGTG CTCAGGCCTG GGCTTGAGAT

601 AAAATGACCC CTTTAGTCCA AGCATCAAAA CAGACCAAGG TTTCAGGCCC CCTTGCCTTT

661 AAATAGATTT CAGGGATTAT TTTCTCCAGC CCTAGACCAC AGCTGACTCC TCACCGCCTC

721 TCCACGAACA GACCTCAGAG TTTTGTTTTC TCTGTCTCTC TCCTTTCTTT CTCCTTTATC

781 TCTGTCTACT GAGGTCCTGG CTGTCCCCCT GCCCCACCCT ACCCTATGCT CTTGGGCTTC

841 TGGCCTCATC TCTAACTTAG CTTCTAATTT TTTCTCTTCT TTTCCTTTCT TTTTTTGAAA

901 CAGAGTCTCA CTCTGTCACC CAGGCTGGAG TGTAGTGGCG TGATCTCAGC TCACTGCAAC

CHOP

961 CTCTGCCTCC AGGGTTCAAG CGTTTCTCGT GCCTCAGCCT CCCAAGTAGC TGGGACTACA

1021 GGTGTGCGTC ACCTTGCTCG GCTAATTTTT ATGTTTTCAG TAGAGACGGG GTTTCACCGT

ATF4

1081 GTTGGCCAGG CTGGTCTCAA ACTCCGGACC TCAGGTGATC CACCTGCCTA GCCTCCCAAA

1141 GTGCTGGGAT TACAGGCATG AGCCACCACG CCCGGCCTAA CTTAGCTTCT AATTCTAATG

ATF6

1201 CCTGGTGAAC CTCTTAAATT TTTTTTTCCC AAGACAAAGT CTCACTCTGT TGGCCAGGCT

1261 GGAGTGCAGG GGTGTGATCA TAGCTCACTG CAGACTCTAA CTCCTGGACA CAAGAGACCC

1321 TCCCATCTTG GCCTCCCAAA GTGCTGGGAT TACAGGCGTG AGCCACCATG CCTGGCCTGC

1381 CTAGTGAACT TGAAGGTCTA GCTTGCGGCA CAGGCTCATG GCATGCACTT AACAGATACG

1441 GAATAAATGG ATGAATGGAA AAAGCCCTGG ACTGGGAATA GTAAACCTGG TAACCAAACC

1501 CAGCTCTGAC CCTGACCTGT AAGTAACTAC TATCTCTGGC CTTGGTGTAC CCCATGTATA

1561 GTGGGGATTG TAAACACCTG CCCTGCCCAC CTTATGATAC TGCTGTGAGA CTCAAAAGAC

1621 ATCATTAGCT CTTAGGCACA GGGAGCTTGG AGGTTAAATC CAATTTGTTT AGTTTTCAAC

1681 AAGGAAGTGG TGCCCCAGGG ACAATGATGG AGAGAAATCG AATGTAATGA GCTGTTGCCA

1741 CCAGCTGAGT GGTCCTGAAA TCATGGCATC TGGGTTGCAC TTAGAGATTG TCCAGTTCAA

1801 AAGGCCAGAA GGAATCTGAA CCTCGATGGG AAAAGTGACT TGCTCAGCTC CCCAGCAAGC

1861 CAGGGCAGAG CTGGAGGATG GACTGGAGTC TCCTGGTTCC AGGTCCAGGA CTTCTTTCCG

1921 CTGTGTTGAC AGAGCCAGGA GGAGGGGCAC CCGGGGAGCA GGGGAGGCAA GGGCTGCTGG

1981 ATGCCCCATC TCAGTTGAAT TCTCCTTGAG GGACCCAGCC CAGGAGCAGA GTAAGAGGGT

2041 TTGAGGGTGG AAGGTGGCAG GGCTGGCCAA GCAGTATAGT CCCTGCTGCT GATAACCAAT

2101 CCCTGAAATC CCGAGGTGGA GGGACTTGAG GGCAAATCAC AGACATGGGG GACCTAATGC

2161 TGGCCATGTG GCTCAACCAG AAGTGCCCGT CCCTCATAGC TTTGGGGAGA TCAGAAGTCA

2221 GGGATAGGGT CGGGTGTGGT GGCTCATGCC TGTAATCCCA TCACTTTGGG ATAAGGAGGC

2281 AGGGGGATCA CTTGAGCTTA GGAGTTCGAG ACCAGCCTGA GCAACATAGC AAAACCCTGT

2341 TCTTTACAAA AAAATACAAA AATTAGCCGG GCGTGGTGGT GCACACCTGT AGTCCTGGCT

ATF6

2401 GCTGGGGAGG CTGAGGTGGG AAGATCACTT GAGCCCGGGA GGTCGAGGCT GCAGTGAGCT

2461 GACATCATGC TACTGCATTC CAGCCTAGGC AATAGAGTGG GACCCTGTCT CAAAAAAAAA

ATF4

2521 AAAAAAAAAA AGTCAGGGGT GCTGGCCCCA TGATAGGTGC AATGGGTGCC TCCAATTCCC

2581 TCTGGCTCTG CCTCCCAGCC TCTGTCCAAG CAACAGGCAG ATTTTCCATG CCTGGGGACT

2641 TGCCCCTGGC TCACTGATGA TGATACCATC TTAAGTCTCC TGGAATCCTT AAACCCTTCC

2701 TTGGCATTTG TGAGCAAGTA TTGAGCCCCT CCCATGTTTT AGGCCCAGTG CTGGGTGCTT

2761 TCACCTGCAA TTTCTTACAG AGGATGGTAT TTCCTAAACT CCATTCATTT GCTACCTCCA

2821 TGATTTTTGC CATATCACCC AACCCATGAA ATACATAAAT AAATAGTATT TACTCAATAT

2881 TTTGACTCAC TTTTAAAAAA CATAAACTTA TCTGAAAAGG GAACCTATGT CACGCCTCTA

2941 AATAAACAGT ATCAACCAAA AATACAAAAG AAACAAAAAT AAATACAAAG ACATTCTTGG

3001 TTTCTGGCAC CAGGCCCCAG TTCTGGCTCC AACCTTACTG AGAAGTCACA TGATCTCTCT

3061 GGGCCTCAGT TTTCTCCTCT GGAAAATGGA GCTTTTGGAA GTTAATGCAT GCACAGTGCC

3121 TGGCCCTGAG ACTGGCATGG AGTGAGTGGA AGAGAGGTTG CCTGACCTGG CTAAAATTGG

3181 TTCTCTGGGC AGACATTTTC CCAAGGGCCA CTGAGAAGAC CCTCCTGTTA GGAGTCAGTG

3241 AGCTCTGTCG CTGGAGGCAT TCAAACAGAG CCTGCGGACT TCTCACTGCG AAGTTGCCCA

3301 GAGAATTCAG TGCTCAGAGG AAAGGGAGTG GTTATTCCAT CAGAGCTGGT TCCCCAGGAG

3361 CGGGAGCAGG GCCTGTAGCA CCCAGCCTCT GTCCCTGGCT CCCGTCTATC CGGGATTTTA

3421 GCGTTTCCTC TGTAGTTTTC AAGCACTGTC TCATATGACT CTCGCACCAG CGAGAGGCCA

3481 GGGGAGATGG TGTCTGCCTG TTAAAGAGGG GCAGGCTGGT CCACATAGGT CAAGTGACTT

3541 GGCCAAGGTC ACCAGAGCAG AGTTTTGAAC TTGAGCTGTC TGACTCAACT GCCTGGGAAG

3601 TGCCTGCCCC TCCTCTGGCA TCCAGGGAGC ATGTCCTGGG GCTCTGGCTG GGACATAGCC

3661 GGACACCTGC GGGCCCTTTA CGTCTCTAAA GAGAGAAAGA GGGAAGGGCC CCTGTCTAGG

3721 GGGTGGTTTC CCTCCAGATG CCACCCCTCC GAGGTCCCCT TCTGCTTCTT CTTGCGGTAG

3781 CCAGGGAGGC AGCCCACGGG CAGGGAAGCG GGGGTGGGGG TGCAGAGTCA GAGGTGCACG

XBP1

3841 TGGACAGAGA CAGAGAGACA GGGACACGTG GGCAGAGACG GATAAAGACA GAGACCCAGA

XBP1

3901 GAAAGCCAGA TATGTTGACA GACACAGAGA CAGACGCCAG AGAGGAAGGC AGACAAAGAG

3961 ACGGGTGGAG ACAAAGACTC CCACCAAGAG ACGCAGAAGG AAGATGCCGA CGGTAAAGAC

4021 AAAACAGGAG ACGCGCGCAA GGAGCAGGTC AGAGCCCAGG CTCGCTGAGA GAGGAAGGGC

4081 TGGGCTGGGG CAGCCCGGAG GCAGAGAGAC CGAGAGTGCG GGGCGGGCGA GGGACGCCAG

4141 GGCCGCGTCA CCCCAGCCCG TTCCTAGCTC CGCTCCCGGC AGGGGGCGCC CTGGCCTCGT

4201 GGCACGACCG GCCCGCGGGG CGCGGGGCTC GGGCCGGGGG CGGGGCCGGG GCCGGGCTGG

4261 GGAGGGGTTG GGGCCGGGGG CGGGGGAGGG GGCGGGCTGC CCGGGCCTCA CTCGGGCCCC

4321 GCGGCCGCCT TTATAAGGCG GCGGGGGTGG TGGCCCGGGC CGCGTTGCGC TCCCGCCACT

TSS

4381 CCGCGCCCGC TATCCTGGCT CCGTGCTCCC ACGCGCTTGT GCCTGGACGG ACCC

**Additional file 1: Figure S1. The sequence of the ALP promoter with ER stress-related transcription factor binding sites.** Binding sites were annotated based on findings from Matinspector (Genomatix).

**Additional file 1: Figure S2. Coomassie gel used for proteomic analysis of proteins bound to the ALP promoter.** Nuclear extracts were bound and eluted off the biotinylated DNA on beads and analysed by SDS-PAGE. Negative control - extracts incubated with beads without biotinylated DNA. Red boxes indicate fragments of gel that were excised and analysed by LC-MS/MS. The lane fragments were selected based on visible differences in bands (indicated with arrows). The molecular weights of relevant ER stress transcription factors are as follows: ATF4 – 38.6 kDa, ATF6 75 kDa, CHOP -19.2 kDa, XBP1 – 28.7 kDa, CREB/ATF – 37 kDa, and with the exception of CHOP would have been encompassed by this analysis.

**Additional file 2. Results of proteomic analysis.** Nuclear extracts were bound and eluted off the biotinylated DNA on beads and analysed by LC-MS/MS. Raw mass spectrometry data were analysed in Proteome Discoverer (ThermoScientific; v1.3.0.339) utilising the Mascot database. Samples were searched against Uniprot database to identify proteins bound to the ALP promoter and compared with proteins that nonspecifically bound to unconjugated beads.

**Additional file 1: Materials and methods**

**Cell culture and treatments**

Human primary VSMCs were of aortic origin, collected from adult organ transplant donors, characterised and archived in the laboratory. All human materials were handled in compliance with the Human Tissue Act (2004, UK). VSMCs were maintained in M199 (Sigma) supplemented with 20% FBS (Gibco) and 100 U/ml penicillin, 100 U/ml streptomycin and 0.29 mg/ml glutamine (Gibco), in 4% CO_2_ at 37°C. Cells were split 1:2 upon reaching 80% of confluency and used for experiments between passages 8-12. For treatments, VSMCs were washed with EBSS 24-48 hours after passaging and incubated in the presence of 0.2 μg/ml thapsigargin (TG, Sigma, T9033) or 0.4 μg/ml Tunicamycin (TM, Sigma, T7765) in M199 supplemented with 0.5% FBS. Stock solutions of TM and TG (1 mg/ml in DMSO) were stored in -20°C.

**Transient gene knock-down with siRNA**

SiRNA transfection was performed in order to knock down ATF4 in VSMCs. Cells were incubated in complete medium for 24 hours and then transfection medium was applied for 48 hours. For one well of a 48 well plate the transfection medium contained 250 μl M199 with 20% FBS and PSG, 25 μl OptiMEM (Gibco), 3 μl HiPerfect (Qiagen) and 3 pmol siRNA oligonucleotide smartpool (GE Dharmacon).

**Quantitative real-time PCR**

RNA was extracted from cells using RNA-STAT 60 (Amsbio) according to manufacturer's protocol. Concentration and purity of isolated RNA was determined using a Nanodrop ND-1000 spectrophotometer. Reverse transcription (RT) was carried out using 0.1 μg/μl RNA, with 7.5 ng/μl of Random and Oligo dT primers (Promega), dNTP mix (0.25 mM each nucleotide, Eurogentec), 0.5 U/μl RNAsin RNase inhibitor (Promega), 5x MU-MLV buffer and 1 U/μl Mu-MLV reverse transcriptase (Eurogentec). Expression of all genes was quantified using the 2^-ΔΔCt^ method and primers (ALP F: ACGAGCTGAACAGGAACAACGT R: CACCAGCAAGAAGAAGCCTTTG, GAPDH F: CGACCACTTTGTCAAGCTC R: CAAGGGGTCTACATGGCAAC) were validated for this method. QPCR was carried out with 2x MESA GREEN qPCR MasterMix and a final concentration of 0.125 μM of each primer in a Corbette RotorGene3000.

**Alkaline phosphatase activity assay**

Alkaline phosphatase (ALP) assay is a colorimetric test based on the hydrolysis of phosphate group from a colourless substrate p-nitrophenyl phosphate (pNPP) by ALP yielding coloured p-nitrophenyl (pNP) product. Cells were scraped with 1% Triton X-100 in PBS and lysed by freeze-thawing twice. The lysates were centrifuged at 13,000 g for 5 minutes at 4°C and supernatants transferred to fresh tubes. Equal volumes of each sample were loaded in triplicate into a 96-well plate. 100 μl of 1 mg/ml pNPP (Sigma) in ALP assay buffer (0.1 M glycine, 1 mM MgCl_2_, 1 mM ZnCl_2_, pH 10.4) was added to each well. The plate was incubated at 37°C for 30 minutes in darkness. Serial dilutions of 10 mM pNP were prepared in the same plate (to form a standard calibration curve) and absorbance was measured at 405 nm. Protein concentration in each sample was measured using the DC Protein Assay according to the manufacturer’s protocol. Alkaline phosphatase activity was normalised to protein concentration in each sample and was expressed as μM of pNP generated per minute.

**Bioinformatic analysis of ALP promoter DNA sequence**

The sequence corresponding to the -122 to -4556 (counting from the first nucleotide of the initiation codon) of the ALP promoter was analysed with Matinspector (Genomatix) for the presence of transcription factor binding sites.

**Luciferase reporter assay**

Ten deletion constructs containing fragments of the human tissue-nonspecific alkaline phosphatase (ALP) promoter cloned into the pGL3-BV vector with the firefly luciferase gene were kindly provided by Dr Hideo Orimo from Nippon Medical School in Tokyo, Japan. The pRL-TK renilla luciferase control vector was kindly provided by Dr Alison Brewer from the Cardiovascular Division, King's College London, UK. The plasmids were transformed into E. coliDH5α and isolated using the NucleoBondXtra Midi plasmid purification kit (Machery-Nagel) according to the manufacturer's protocol. VSMCs were co-transfected with the pGL3-BV ALP promoter constructs and pRL-TK using Lipofectamine LTX. 24 hours after transfection cells were washed and treated with TM and TG, as described previously, for 24 hours. Luciferase assays were carried out using the Dual-Luciferase Reporter Assay kit (Promega) according to the manufacturer's protocol. Firefly luciferase signal was normalized to renilla luciferase signal.

**Nuclear extraction**

Cells were plated in a T25 flask (100 000 cells per flask) and treated as described. Then cells were washed with cold PBS/phosphatase inhibitor buffer (125 mM NaF, 250 mM β-glycerophosphate, 25 mM NaVO_3_, 1:20), scraped with PBS/PIB and centrifuged at 350 g for 5 minutes at 4°C. The pellet was resuspended in 1ml of hypotonic buffer and incubated 15 minutes on ice to allow cells to swell. Next, 50 μl of 10% NP-40 was added to disrupt the cell membranes without damaging nuclear membranes and samples were thoroughly mixed and centrifuged 1 minute at 14,000 g at 4°C to pellet the nuclei. Then pellets were resuspended in 40μl nuclear lysis buffer (50 mM Tris HCl (pH 8), 150 mM NaCl, 1% NP-40, 0.5% sodium deoxycholate, 1% glycerol, 1 mM NaVO_3_, 2 μl/ml protease inhibitor cocktail) and incubated 30 minutes on ice with gentle rocking. During the incubation the pellets were resuspended every 10 minutes. The samples were then centrifuged 10 minutes at 14000 g at 4°C, after which supernatants containing nuclear proteins extracts were saved. Concentrations of the extracts were measured with DC Protein Assay and they were stored in -80°C.

**DNA binding assay**

A biotinylated oligonucleotide with the promoter sequence was generated with a PCR reaction using a biotinylated forward and normal reverse primers (F: GGAGTGTAGTGGCGTGATCT, R: GCAATAGAGTGGGACCCTGT). The oligonucleotide was then precipitated with 3M sodium acetate (pH 5.5), 2.5 volumes of 95% ethanol for 30 minutes at -20°C. The DNA was pelleted at 14,000 rpm at 4°C for 15 minutes, washed with 70% ethanol and centrifuged again. The pellet was resuspended in double-distilled water and the concentration and purity was determined using the ND-1000 Nanodrop spectrophotometer. For each tested condition 6μg of the oligonucleotide was conjugated with 20 μl of magnetic streptavidin-coated beads (Dynabeads M-270 Streptavidin, Invitrogen). Magnetic beads were washed 3 times with 10 volumes of B&W buffer (10 mM Tris-HCl, pH 7.5, 1 mM EDTA, 2 M NaCl). Biotinylated DNA was added to the beads resuspended in B&W buffer and incubated with rotation 30 minutes at room temperature. After that the beads were washed 3 times with B&W buffer, twice with transcription factor binding buffer and aliquoted into fresh tubes. Competitor DNA poly(dI:dC) was added to each aliquot (5 μg for each 20 μl of beads) and samples were incubated for 15 minutes at room temperature with rotating, to decrease unspecific interactions of DNA with proteins. Next, nuclear extracts (20 μg) were added to each sample and incubated 30 minutes rotating. After that the beads were washed 3 times with transcription factor binding buffer and bound transcription factors were eluted by resuspending the beads in SDS-PAGE sample buffer and boiling 5 minutes at 95°C. Samples were analysed by Western blotting.

**Western blotting**

Samples were analysed on 10% acrylamide gels. Proteins were transferred to PVDF membranes (Immobilon-FL, Milipore) using a Trans-Blot SD Semi-Dry Transfer Cell (Bio-Rad) at 25 V for 1 hour. Membranes were blocked in PBST with 5% milk for 1 hour at room temperature and then incubated with primary antibody (anti-ATF4 Santa Cruz, sc-200; 1:1000) diluted in blocking buffer, overnight at 4°C. IRDye-conjugated secondary antibody (Anti-Rabbit IRDye680 RD, Li-Cor, 926-68071; 1:10000) was applied for 1 hour at room temperature. Bands were visualised with Li-Cor Odyssey CLx infrared scanner.

**Mass spectrometry**

Samples were ran on an SDS-PAGE acrylamide gel. The gel was stained with BioSafe Coomassie (BioRad), fragments of the gel were cut out and in-gel reduction, alkylation and digestion with trypsin were performed prior to subsequent analysis by liquid chromatography - tandem mass spectrometry (LC-MS/MS). Raw mass spectrometry data were analysed in Proteome Discoverer (ThermoScientific; v1.3.0.339) utilising the Mascot database. Samples were searched against Uniprot database to identify proteins.

**Data analysis**

All results represent 3 independent experiments, unless stated otherwise. Data was analysed using Apache OpenOffice 4.1.1 and GraphPad Prism 5 software. Graphs show mean with SEM. Where appropriate t-tests or one way ANOVA with Tukey's or Dunnett's post hoc tests were performed. Results were considered statistically significant when p<0.05. Statistical significance is indicated with asterisks: *denotes p between 0.05 and 0.01, ** denotes p between 0.01 and 0.001, *** denotes p<0.001.
